# Cell Proliferation, Chondrogenic Differentiation, and Cartilaginous Tissue Formation in Recombinant Silk Fibroin with Basic Fibroblast Growth Factor Binding Peptide

**DOI:** 10.3390/jfb15080230

**Published:** 2024-08-17

**Authors:** Manabu Yamada, Arata Nakajima, Kayo Sakurai, Yasushi Tamada, Koichi Nakagawa

**Affiliations:** 1 Department of Orthopaedic Surgery, Toho University Graduate School of Medicine, 5-21-16 Omori-nishi, Ota-ku, Tokyo 143-8540, Japan; manabu.yamada@med.toho-u.ac.jp; 2 Department of Orthopaedic Surgery, Toho University Sakura Medical Center, 564-1 Shimoshizu, Sakura, Chiba 285-0841, Japankonakag@med.toho-u.ac.jp (K.N.); 3 Department of Rehabilitation, Toho University Sakura Medical Center, 564-1 Shimoshizu, Sakura, Chiba 285-0841, Japan; 4 Faculty of Textile Science and Technology, Shinshu University, 3-15-1 Tokida, Ueda, Nagano 386-8567, Japan; ytamada@shinshu-u.ac.jp

**Keywords:** fibroin sponge, P7 peptide, basic fibroblast growth factor (bFGF), mesenchymal cell, proliferation, cartilage

## Abstract

Regeneration of articular cartilage remains a challenge for patients who have undergone cartilage injury, osteochondritis dissecans and osteoarthritis. Here, we describe a new recombinant silk fibroin with basic fibroblast growth factor (bFGF) binding peptide, which has a genetically introduced sequence PLLQATLGGGS, named P7. In this study, we cultured a human mesenchymal cell line derived from bone marrow, UE6E7-16, in wild-type fibroin sponge (FS) and recombinant silk fibroin sponge with P7 peptide (P7 FS). We compared cell proliferation, chondrogenic differentiation and cartilaginous tissue formation between the two types of sponge. After stimulation with bFGF at 3 ng/mL, P7 FS showed significantly higher cell growth (1.2-fold) and higher cellular DNA content (5.6-fold) than did wild-type FS. To promote chondrogenic differentiation, cells were cultured in the presence of TGF-β at 10 ng/mL for 28 days. Immunostaining of P7 FS showed SOX9-positive cells comparable to wild-type FS. Alcian-Blue staining of P7 FS also showed cartilaginous tissue formation equivalent to wild-type FS. A significant increase in cell proliferation in P7 FS implies future clinical application of this transgenic fibroin for regeneration of articular cartilage. To produce cartilaginous tissue efficiently, transgenic fibroin sponges and culture conditions must be improved. Such changes should include the selection of growth factors involved in chondrogenic differentiation and cartilage formation.

## 1. Introduction

Regeneration of articular cartilage remains a challenge for patients with cartilage injury, osteochondritis dissecans and osteoarthritis. To date, many approaches to solving this problem have been investigated. Among them, the local transplantation of cells with biomaterials has been tested in experimental animal models of articular cartilage defects [1,2,3,4,5,6]. At present, autologous chondrocyte implantation is generally applied to limited cartilage defects of the knee [7,8]. However, treatment of large defects of articular cartilage has not been established.

In animal models, several types of scaffold have been combined with mesenchymal stem cells to test their efficacy in repairing articular cartilage defects. Thus far, inclusion of a scaffold has had limited benefits in the regeneration of articular cartilage [9,10,11,12,13,14,15]. The most serious issue has been inadequate strength to support physiological loads applied to articular cartilage. Fibroin sponge has been reported as a scaffold material with suitable mechanical properties matching those of articular cartilage. In addition, fibroin has low inflammatory properties [16] and provides good attachment, benefiting cell viability, growth and function [17]. Previously, we collected chondrocytes from Japanese white rabbits and cultured cells with silk fibroin sponge (FS). We found that well-defined cartilage tissue was produced in FS [18]. Furthermore, in osteochondral defects of the rabbit patella, wrapping with FS containing chondrocytes successfully produced hyaline-like cartilage [19]. These findings suggest that FS covered with cultured cells, such as chondrocytes, has the potential to support the repair of large osteochondral defects.

To use such tissue engineering technology for the treatment of large osteochondral defects, increasing the number of cells cultured in FS is quite important because adult human cells generally have limited ability to proliferate. Among cell growth factors, basic fibroblast growth factor (bFGF) has potent mitogenic effects [20]. To increase the proliferative activity of cells seeded in FS, we previously generated transgenic silkworm fibroin fused to basic fibroblast growth factor (bFGF). Chondrocytes seeded in bFGF-fused FS showed higher cell growth activity than did wild-type. However, those in bFGF-fused FS showed lower expression levels of type II collagen than did wild-type [21]. A possible reason for the limited usefulness of bFGF-fused FS was that the conformation of genetically produced bFGF was unstable and denatured during processing. To overcome this issue, we recently developed recombinant silk fibroin combined with basic fibroblast growth factor (bFGF) binding peptide [22]. This approach has considerable potential for therapeutic application because a silk fibroin matrix can be combined with bFGF in amounts appropriate for timed release. This newly developed matrix could be applied to tissue defects or wound areas, thereby utilizing the patient’s own bFGF, leading to rapid tissue construction or wound healing.

In this study, we cultured a human mesenchymal cell line derived from bone marrow, UE6E7-16, with wild-type or recombinant silk fibroin with bFGF-binding peptide. We compared cell proliferation, chondrogenic differentiation, and cartilaginous tissue formation between sponges.

## 2. Materials and Methods

### 2.1. Preparation of Silk Fibroin Sponge

Silk fibroin sponge (FS) was processed as described previously [18]. In brief, an aqueous solution of silk fibroin was prepared by dissolving silkworm (Bombyx mori) cocoons in 9M LiBr, followed by dialysis against pure water. FS was formed as a result. The aqueous solution of silk fibroin (4 *w*/*v*%) was mixed with dimethyl sulfoxide (DMSO) at 1% *v*/*v* and was frozen at −20 °C followed by thawing. After removal of DMSO, FS was sterilized by autoclaving. This sponge was designated as wild-type FS.

**Figure 1:**
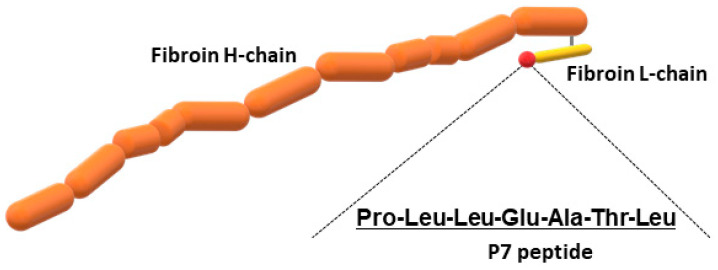
Schematic drawing of recombinant silk fibroin fused to P7 peptide.

**Figure 2:**
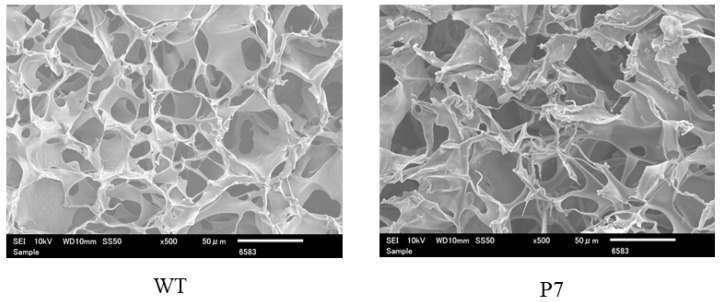
Scanning electron microscopic images of wild-type (WT) and P7 fibroin sponge. P7 fibroin sponge (P7) is a recombinant silk fibroin with bFGF-binding peptide, named P7. Scale bars = 50 µm.

In addition to wild-type FS, we prepared recombinant silk fibroin with bFGF-binding peptide, which has a genetically introduced sequence PLLQATLGGGS, named P7. The oligonucleotide sequence encoding P7 was inserted at the C-terminus of the fibroin L-coding sequence (Figure 1). The presence of P7 peptide in the recombinant fibroin molecules was confirmed by ELISA [22]. Since P7 peptide has high homology to the immunoglobulin-like domain III of bFGF receptors [22], P7-fused FS (P7 FS) was expected to increase cultured cell growth and promote tissue regeneration. Images of wild-type and P7 FS were obtained by scanning electron microscopy and are shown in Figure 2. For cell culture experiments, FS was shaped into disk form in 6 mm Φ and 1.5 mm thicknesses.

### 2.2. Cell Culture and Chondrogenic Differentiation

**Figure 3:**
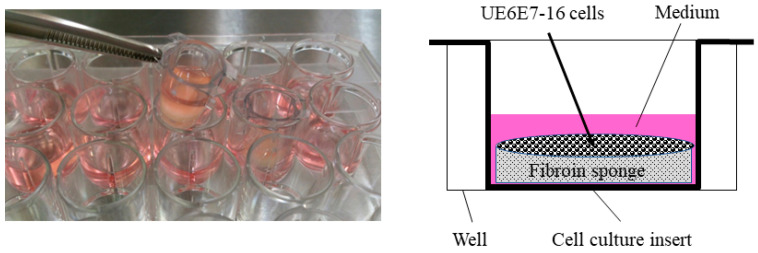
Cell culture in fibroin sponge using a cell culture insert. Cells were cultured in the presence of bFGF for 3 days and subjected to a cell proliferation assay. Following stimulation with bFGF, cells were also cultured in the presence of TGF-β3 for 28 days to evaluate chondrogenic differentiation and cartilaginous tissue formation in the sponge. bFGF, basic fibroblast growth factor; TGF, transforming growth factor.

A human mesenchymal cell line derived from bone marrow, UE6E7-16 (RCB2163), was purchased from RIKEN BioResource Research Center (Tsukuba, Japan) and used in this study. For cell proliferation assays, cells were cultured in POWEREDBY10 (GlycoTechnica, Yokohama, Japan) for 1 day, then seeded in FS (1.0 × 10^6^ cells/sponge) in DMEM containing 0.5% FBS. To stabilize FS in the medium, a cell culture insert (CORNING, Corning, NY, USA) was placed in a 24-well plate (Figure 3), where cells were cultured for an additional 3 days in the presence or absence of bFGF (KAKEN Pharmaceutical Co., Ltd., Tokyo, Japan).

For chondrogenic differentiation, cells were cultured in POWEREDBY10 for 1 day, then seeded in FS (1.0 × 10^6^ cells/sponge) in DMEM containing 0.5% FBS, using a cell culture insert. The cells were further cultured with a human mesenchymal stem cell chondrogenic differentiation medium bullet kit (Lonza, Walkersville, MD, USA) in the presence of TGF-β3 (Lonza) at 10 ng/mL for up to 28 days. Basic FGF at 3 ng/mL was added only for the first 3 days. The medium containing the growth factors was changed every day throughout the culture period.

### 2.3. Cell Proliferation Assay

On day 3, cells were subjected to proliferation assays using a cell counting kit-8 (CCK-8, Dojindo, Kumamoto, Japan). Briefly, cells were incubated for an appropriate length of time in the incubator at 37 °C, after which the supernatant was transferred to a 96-well plate. After adding CCK-8 solution to each well of the plate, the absorbance at 450 nm was measured using a microplate reader.

Cells were also subject to quantification of genomic DNA using a DNA extraction kit according to the user manual (NucleoSpin Tissue, MACHEREY-NAGEL, Duren, Germany). Before using the kit, the FS was minced with scissors and stirred in a solution using a vortex, followed by cell collection. Three to four sponges were tested in each experimental condition.

### 2.4. Chondrogenic Differentiation and Cartilaginous Tissue Formation

On day 28, FS was subjected to histological analyses. FS was fixed with 10% formalin and embedded in paraffin. Midsagittal sections 6 μm thick were mounted on silane-coated slides. Sections were reacted with a rabbit monoclonal antibody against Sox9 (EPR14335, Abcam, Cambridge, UK, 1:2000). Immunostaining was performed as previously described [23]. Signals were detected using diaminobenzidine (DAB). For the negative control sections, the same procedures were used except that the primary antibody was replaced by nonimmune rabbit immunoglobulin G. Four regions (0.3 mm × 2.4 mm in size) containing FS and seeded cells were analyzed in each section. The number of Sox9-positive cells and total cells was counted, and the percentage of Sox-9 positive cells was calculated. Three to four sponges for wild-type and P7 FS were evaluated.

To evaluate cartilaginous tissue formation, sections were subject to Alcian-Blue staining. The blue-stained areas in fibroin sponges were defined as cartilaginous tissue.

### 2.5. Statistical Analyses

The significance of differences between groups was assessed by *t*-test, Mann–Whitney’s U-test, or one-way ANOVA. A value of *p* < 0.05 was considered statistically significant.

## 3. Results

### 3.1. Proliferation of UE6E7-16 Cells Cultured in FS

**Figure 4:**
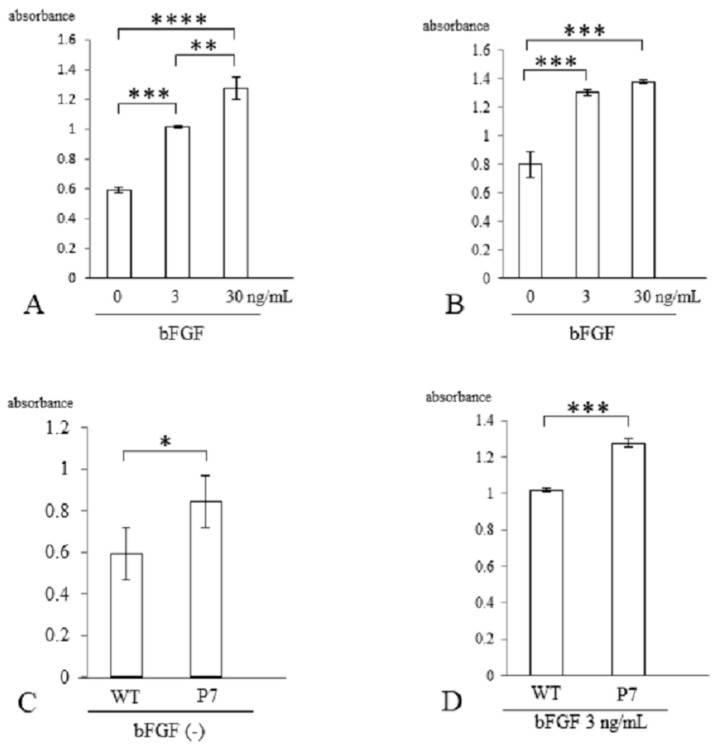
Cell proliferation of UE6E7-16 cells cultured in FS for 3 days. (**A**) Wild-type FS. Stimulation with bFGF increased cell growth in a dose-dependent manner. (**B**) P7 FS. Stimulation with bFGF increased cell growth but not in a dose-dependent manner. (**C**) Comparison between wild-type and P7 FS without stimulation of bFGF. (**D**) Comparison between wild-type and P7 FS after stimulation with bFGF. Error bars show standard deviation. Significant difference, * *p* < 0.05, ** *p* < 0.01, *** *p* < 0.005, **** *p* < 0.001. WT, wild-type; FS, fibroin sponge; bFGF, basic fibroblast growth factor.

Three days after stimulation with bFGF, cell proliferation was assessed with a CCK-8 kit. In wild-type FS, cell growth was significantly elevated with bFGF at 3 ng/mL (1.7-fold, *p* < 0.005) and 30 ng/mL (2.2-fold, *p* < 0.001) compared to cells without stimulation. There was also a significant difference between 3 and 30 ng/mL (*p* < 0.01) (Figure 4A). In P7 FS, cell growth was significantly elevated with bFGF at 3 ng/mL (1.6-fold) and 30 ng/mL (1.8-fold) compared to cultures lacking stimulation (*p* < 0.005). However, no significant difference was detected between 3 and 30 ng/mL (Figure 4B).

When comparing wild-type and P7 FS after 3 days of cultivation, P7 FS showed significantly higher cell numbers (1.4-fold) than wild-type FS (*p* < 0.05) (Figure 4C). After stimulation with bFGF at 3 ng/mL, P7 FS showed significantly higher cell growth (1.3-fold) than wild-type FS (*p* < 0.005) (Figure 4D).

### 3.2. Measurement of DNA Content of UE6E7-16 Cells Cultured in FS

**Figure 5:**
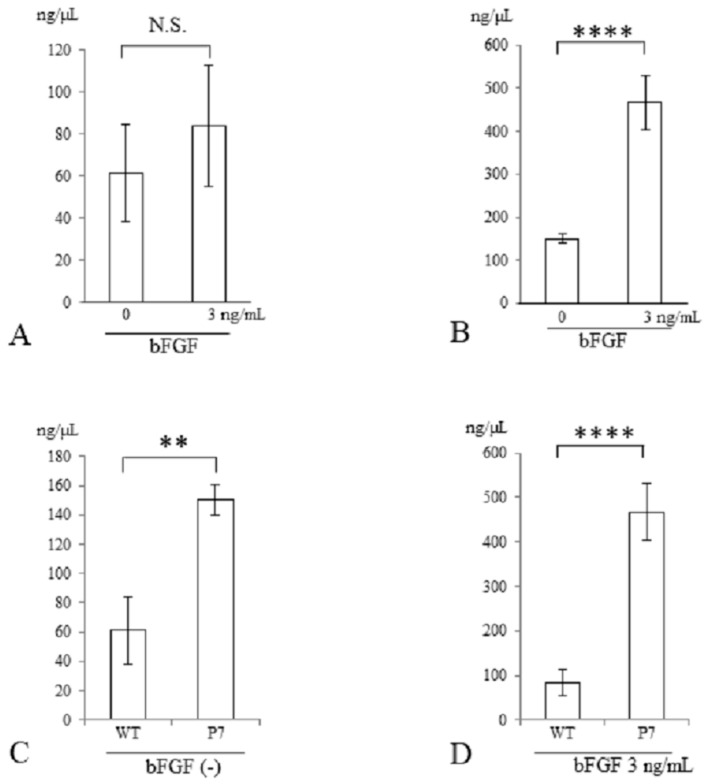
DNA quantification of UE6E7-16 cells cultured in FS for 3 days. (**A**) Wild-type FS. (**B**) P7 FS. (**C**) Comparison between wild-type and P7 FS without stimulation of bFGF. (**D**) Comparison between wild-type and P7 FS after stimulation with bFGF. Error bars show standard deviation. Significant difference, ** *p* < 0.01, **** *p* < 0.001. N.S., not significant; WT, wild-type; FS, fibroin sponge; bFGF, basic fibroblast growth factor.

Three days after stimulation with bFGF at 3 ng/mL, the genomic DNA content of cells seeded in FS was measured. In wild-type FS, the total DNA contents were similar in cultures with and without bFGF stimulation (Figure 5A). In contrast, in P7 FS, they were significantly elevated with bFGF stimulation compared to those without stimulation (3.1-fold, *p* < 0.001) (Figure 5B).

In a comparison between wild-type and P7 FS after 3 days of cultivation, P7 FS showed significantly higher DNA content than did wild-type FS (2.5-fold, *p* < 0.01) (Figure 5C). After stimulation with bFGF at 3 ng/mL, P7 FS showed significantly higher DNA content than did wild-type FS (5.6-fold, *p* < 0.001) (Figure 5D).

### 3.3. Chondrocyte Differentiation of UE6E7-16 Cells in FS

**Figure 6:**
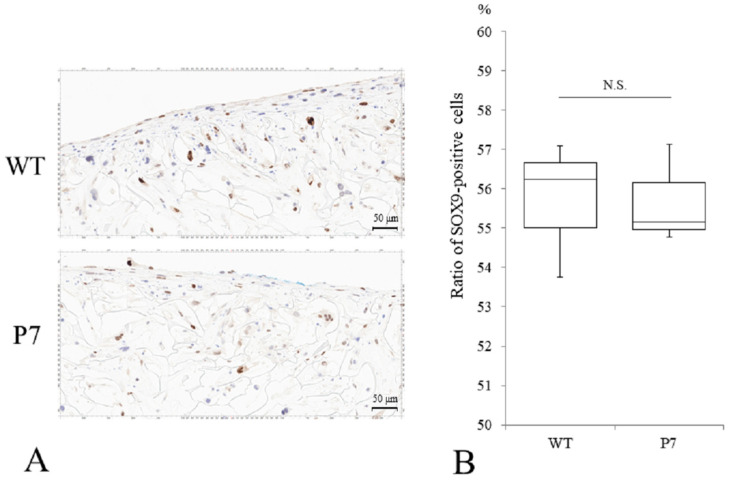
Chondrogenic differentiation of UE6E7-16 cells cultured in FS for 28 days. (**A**) Immunostaining of SOX9 for WT and P7 FS. Scale bars = 50 µm. (**B**) A median ratio (%) of SOX9-positive cells to total cells. Thick horizontal lines, boxes, and whiskers show median, interquartile range (IQR), and most extreme points from the limits of the box, respectively. N.S., not significant; WT, wild-type; FS, fibroin sponge.

Immunostaining of P7 FS showed SOX9-positive cells comparable to that seen in wild-type FS (Figure 6A). The median ratio of Sox9-positive cells to total cells was 56.3% for wild-type and 55.2% for P7, respectively, with no significant difference (Figure 6B).

### 3.4. Cartilaginous Tissue Formation in FS

**Figure 7:**
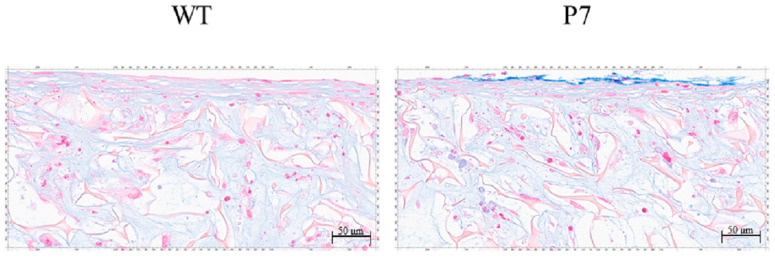
Cartilaginous tissue formation by UE6E7-16 cells cultured in FS for 28 days. Cartilaginous tissue is rendered light blue with Alcian-Blue staining. P7 FS shows an equivalent amount of cartilaginous tissue to that seen in wild-type FS. Scale bars = 50 µm. WT, wild-type; FS, fibroin sponge.

In both wild-type and P7 FS, cartilaginous tissue, which was rendered light blue by Alcian-Blue staining, was formed among fibroin fibers in the superficial layer of the sponges. P7 FS showed an equivalent amount of cartilaginous tissue to that seen in wild-type FS (Figure 7).

## 4. Discussion

In the present study, we analyzed cell proliferation and cartilaginous tissue formation by a human mesenchymal cell line, UE6E7-16, cultured in P7 FS, with comparisons to wild-type FS. The results show that P7 FS significantly increased cell growth compared to wild-type; however, chondrogenic differentiation and cartilaginous tissue formation were comparable.

We previously showed that proliferation of primary articular chondrocytes in fibroin sponges was significantly increased on bFGF-fused fibroin sponges compared with wild-type. In contrast, the expression levels of a gene encoding collagen type II α 1 chain were lower in bFGF-fused FS than wild-type [21]. This observation supports our present results showing increased cell growth but not enhanced cartilaginous tissue formation in P7 FS, although the cells and fibroin sponges used were different from those previously investigated [21].

Silk fibroin sponges have a mechanically stable and porous structure [24,25,26]. Consequently, it is expected that they could be used as a scaffold source material for musculoskeletal tissue regeneration, including bone, tendon, ligament and articular cartilage [27]. Since treatment of large defects of articular cartilage is clinically challenging, we investigated cell proliferation, chondrogenic differentiation, and cartilaginous tissue formation in FS. When using bio-synthetic materials for the treatment of large tissue defects, it is important to use materials loaded with a large number of cells. We used a human bone marrow-derived mesenchymal cell line, UE6E7-16, and chose bFGF as a growth factor, as it possesses mitogenic activity for many cell types [28,29,30]. Because bFGF-fused FS had limited effects on cell proliferation and cartilage matrix production [21], we developed P7 FS and characterized cell proliferation, chondrogenic differentiation and cartilaginous tissue formation. In the cell proliferation assay, P7 FS showed a slight increase in the presence of bFGF compared with wild-type FS (Figure 4D). On the other hand, quantification of genomic DNA extracted from cells was markedly increased with bFGF stimulation in P7 FS compared to wild-type FS (Figure 5D). This could be due to differences in measurement methods between WST-8 formazan and DNA content. The former method quantified WST-8 formazan in supernatants whereas the latter quantified DNA content from total cells. Notably, P7 FS showed significantly higher cell growth and greater cellular DNA content than wild-type FS even when lacking bFGF stimulation (Figure 4C and Figure 5C). This may suggest that P7 FS allows efficient binding to low levels of bFGF in the medium.

Previous studies have shown that bFGF inhibited chondrocyte differentiation and cartilaginous matrix production [31,32,33,34]. In terms of cartilage regeneration using scaffolds, Deng et al. used gelatin microspheres loaded with bFGF for controlled and sustained release and stimulated repair of knee cartilage defects in rabbits. Their histological evidence showed that previous cartilaginous defects were filled with hyaline-like cartilage, illustrating the potential of a bFGF scaffold to promote chondrogenesis [35]. Others have reported a positive effect of bFGF on cell differentiation and viability despite bFGF-mediated downregulation of collagen type II mRNA [36].

Human mesenchymal stem cells (hMSCs) undergo multi-lineage differentiation and are capable of rapid proliferation. Given their ready availability, they represent a promising candidate for cartilage tissue engineering [37,38]. To induce chondrogenic differentiation, hMSCs require the appropriate signals. Several previous studies have demonstrated that a variety of growth factors, such as bFGF, bone morphogenic proteins (BMP), insulin-like growth factor-I (IGF-I) and TGF-β, can induce the differentiation of mesenchymal cells to chondrocytes under certain culture conditions [39,40,41,42,43,44]. In the present study, to effectively differentiate UE6E7-16 cells from chondrocytes, we cultured the cells in chondrogenic differentiation medium with 10 ng/mL of TGF-β. However, chondrogenic differentiation and cartilaginous tissue formation in P7 FS were comparable to those observed with wild-type FS. A previous study analyzed the use of extracellular matrix-based hydrogel scaffolds with TGF-β and collagen binding domain-bFGF. They reported that hMSCs differentiated from chondrocytes and the differentiation process could be regulated by the controlled release of growth factors [45]. Taken together with these observations, it is evident that both bFGF and TGF-β play important roles in chondrogenic differentiation of hMSCs, but stimulation with such growth factors needs to be regulated.

In the present study, bFGF was added for the first 3 days, whereas TGF-β was added to the chondrogenic differentiation medium throughout the 28 days culture period. Basic FGF could bind to the P7 peptide and exert its mitogenic ability on the cells. In contrast, TGF-β could not act on the cells efficiently since it was simply added to the medium. Therefore, insufficient TGF-β signaling to the cells seeded in FS may be a possible reason for the lack of enhanced chondrogenic differentiation and cartilaginous tissue formation observed in P7 FS. Given P7 FS’s high binding capacity for bFGF, it is not surprising that it generated more cells than the wild-type FS. However, the cells did not differentiate to chondrocytes or produce cartilage matrices. Presumably, this was due to insufficient TGF-β signaling. To enhance the chondrogenic differentiation and formation of cartilaginous tissue in P7 FS, improvements, such as providing a TGF-β-binding domain or peptide in the fibroin, will be necessary.

In this study, we could not investigate expression levels of genes involved in chondrocyte differentiation due to difficulties in extraction of mRNAs from cells seeded in FS. In addition, production of cartilage matrix was not measured. To better understand differences in chondrocyte differentiation and production of cartilage matrix between wild-type and P7 FS, cartilage-related gene expression analyses or measurements of cartilage matrix such as glycosaminoglycan (GAG) or type II collagen will be needed.

There are some limitations in this study. First, the content of the P7-fused L-chain to total L-chain in transgenic fibroin was approximately 25% [22]. In addition, we did not analyze the in vitro binding ability of bFGF to the P7 peptide. Improvement of transgenic fibroins with a higher ratio of P7-fused L-chain is probably necessary. Moreover, in vitro studies of the binding ability of bFGF to the P7 peptide are crucial for application of this material to future clinical use. Second, we used a human mesenchymal cell line, UE6E7-16, but not MSCs from bone marrow. Considering clinical application of the fibroin sponges, experiments using human MSCs from bone marrow will be indispensable. Third, cells were not seeded in deep layers of fibroin sponges, i.e., most of the cells were observed in the superficial layers of FS. For the treatment of large defects of articular cartilage, technology to allow seeded cells to expand to interior regions of FS is desirable. Nevertheless, P7 FS successfully increased a human mesenchymal cell line, UE6E7-16, compared to wild-type FS, which implies future clinical application of this transgenic fibroin for the treatment of large articular cartilage defects.

## 5. Conclusions

P7 FS increased growth of a human mesenchymal cell line, UE6E7-16, compared with wild-type FS, but it did not promote chondrogenic differentiation or cartilaginous tissue formation. To produce cartilaginous tissue efficiently, improvements of the transgenic fibroin sponges and culture conditions including regulation of growth factors involved in chondrogenic differentiation and cartilage formation are required.

## Data Availability

The original contributions presented in the study are included in the article, further inquiries can be directed to the corresponding author.
